# Supplementary material for: Differential effects of lysophospholipid headgroups, acyl chain length and saturation on vacuole acidification, Ca2+ transport, membrane fluidity, and fusion
Source: Front Cell Dev Biol. 2026 Apr 29;14:1805164. doi: 10.3389/fcell.2026.1805164 (PMC13168183; doi:10.3389/fcell.2026.1805164)
Supplement: Supplementary file 1 [file DataSheet1.pdf]

Supplemental Information

Differential effects of lysophospholipid headgroups, acyl chain length and saturation on vacuole acidification, Ca^{2+} transport, and fusion

Chi Zhang et al.

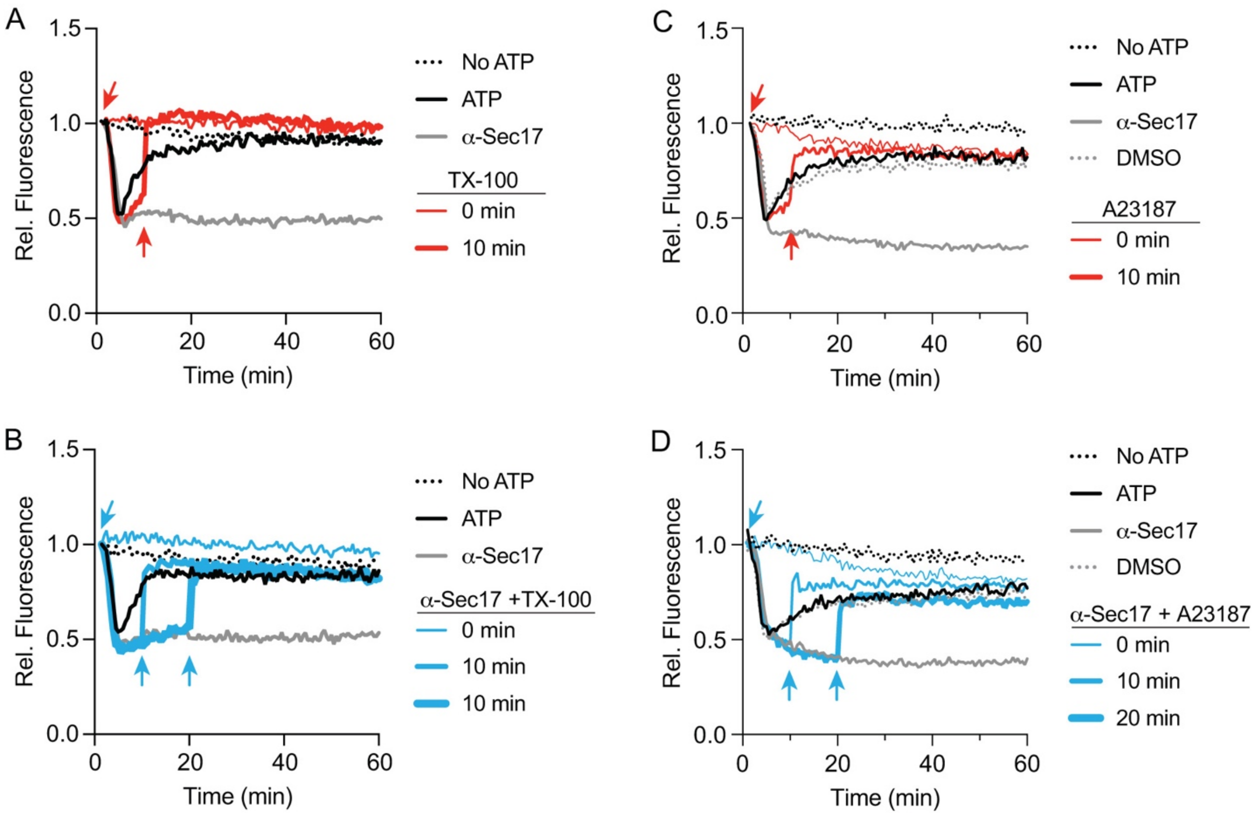

Supplemental Figure 1. Effect of TX-100 and A23187 on Ca^{2+} transport. Vacuoles were isolated from BJ3505 were treated with 140 µg/mL α-Sec17 IgG, 1% TX-100, 100 µM A23187 or PS added at T=0 or 10 min in the presence or absence of ARS and in the presence of Cal520. **(A)** 1% TX-100 was added at T=0 or 10 min (red arrows). **(B)** 1% TX-100 was added to reactions containing anti-Sec17 IgG at T=0 or 10 min (blue arrows). **(C)** 100 µM A23187 was added at T=0 or 10 min (red arrows). **(B)** 100 µM A23187 was added to reactions containing anti-Sec17 IgG at T=0 or 10 min.

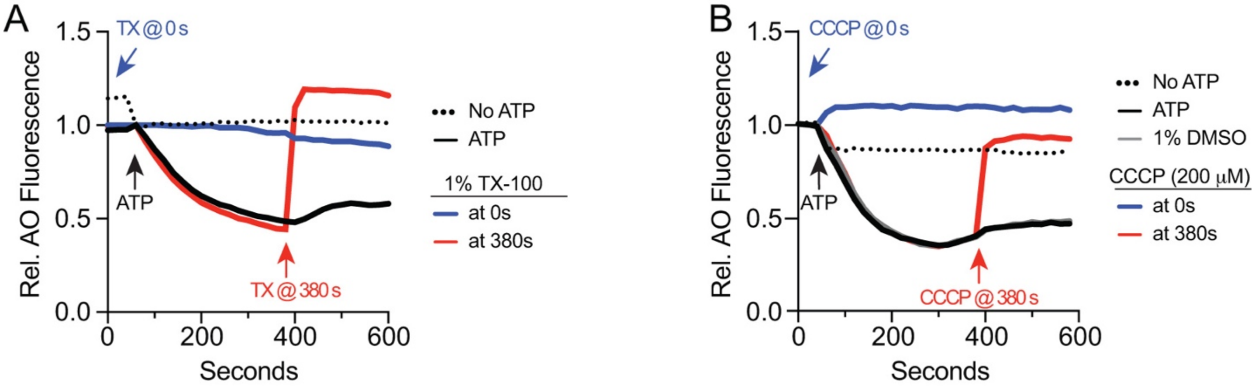

Supplemental Figure 2. Effect of TX-100 or CCCP on vacuole acidification. BJ3505 vacuoles fusion reactions (2X) were incubated with 1% TX-100 **(A)**, 200 μM CCCP **(B)** or reaction buffer at T=0 or 380 sec in the presence or absence of ARS and in the presence of AO. After 60 s of incubation received ARS or additional buffer and further incubated for a total of 800 s. AO fluorescence was normalized to the fluorescence at the time of adding ARS and set to 1.
